# Sex‐specific differences in telomere length of patients with primary knee osteoarthritis

**DOI:** 10.1111/jcmm.18107

**Published:** 2024-01-18

**Authors:** Lukasz Kuszel, Tomasz Trzeciak, Beata Begier‐Krasinska, Magdalena Richter, Jian Li, Malwina Czarny‐Ratajczak

**Affiliations:** ^1^ Department of Medical Genetics Poznan University of Medical Sciences Poznan Poland; ^2^ Department of Orthopedics and Traumatology Poznan University of Medical Sciences Poznan Poland; ^3^ Department of Hypertensiology, Angiology and Internal Diseases Poznan University of Medical Sciences Poznan Poland; ^4^ Department of Biostatistics & Data Science Tulane School of Public Health and Tropical Medicine New Orleans Louisiana USA; ^5^ Department of Medicine, Center for Aging Tulane University, School of Medicine New Orleans Louisiana USA; ^6^ Department of Medicine, Center for Biomedical Informatics and Genomics Tulane University, School of Medicine New Orleans Louisiana USA

**Keywords:** cartilage, leukocytes, primary knee osteoarthritis, relative telomere length

## Abstract

Accelerated telomere shortening is associated with age‐related diseases, including osteoarthritis (OA). We aimed to determine the relative telomere length (TL) in leukocytes and cartilage of patients with primary knee OA and to investigate factors that may affect TL in OA. Relative TL measurements were performed using qPCR in leukocytes of 612 individuals (310 patients with primary knee OA undergoing total knee arthroplasty (TKA) and 302 unaffected controls). We also analysed cartilage in 57 of the 310 OA patients, measuring relative TL in severely affected and less affected (control) cartilage collected from the same knee. Cartilage TLs were compared to leukocyte TLs in all 57 patients. A significant sex‐by‐disease‐status interaction was found in regard to relative TL. Controlling for age, the average difference of leukocyte TL between female OA patients versus female controls was 0.217 units greater than that between male OA patients versus male controls (95% CI; [0.014, 0.421]). Relative TL comparison of severely and less affected cartilage samples from the same joint showed attrition of telomeres corresponding to disease severity (0.345 mean TL difference with 95% CI of [0.151, 0.539]) in the joint. We also noted that both severely and less affected cartilage had shorter telomeres than leukocytes collected from the same patient. Severe and moderate pain in OA patients was associated with shorter TL in leukocytes, but there was no association with depression or smoking in leukocytes and cartilage. Our study indicates that sex is an important factor in OA contributing to leukocyte and cartilage TL and that pain in OA shows an inverse association only with leukocyte TL.

## 1 INTRODUCTION

Although its direct cause is still unknown, overlapping genetic, epigenetic and environmental factors are considered significant contributors to primary osteoarthritis (OA).^1^ The pathogenesis of secondary OA is well understood: It is caused by trauma, bone dysplasias, and metabolic and hormonal disorders, among other conditions.^2^, ^3^

Understanding the genetic predisposition to primary osteoarthritis (OA) has been the subject of extensive research, with several candidate genes and genetic variants identified as potential contributors.^4^, ^5^, ^6^ Despite these advances, the precise molecular mechanisms driving primary OA remain elusive, with recent attention turning to the role of epigenetic changes involved in disease pathogenesis. Among these, telomere shortening has emerged as a key epigenetic factor, with implications in cellular aging and OA aetiology. This study focuses on the extent of telomere attrition in leukocytes and cartilage of OA patients, seeking to elucidate a role of sex and tissue type in OA‐associated telomere shortening.

Accelerated telomere shortening is associated with many ageing‐related diseases, including osteoarthritis.^7^, ^8^, ^9^, ^10^ Telomere shortening of 50–150 base pairs (bp) occurs naturally with every division of the cell, since the linear structure of DNA ends cannot be fully replicated.^11^, ^12^ This process is considered a hallmark of ageing, and cellular senescence is triggered when telomeres reach the critical length threshold (the Hayflick Limit).^13^ Telomere shortening can be accelerated by many factors, including oxidative stress, smoking, high BMI, psychological stress (in both adults and newborns whose mothers have been exposed to stress during pregnancy) and other factors.^14^, ^15^ The minimal length of telomeres in human leukocytes to maintain chromosome stability is 3.81 kb.^15^ Chondrocytes and other somatic cells are devoid of telomerase activity, which can elongate telomeres and is detected in stem cells and germ cells.^14^

Immune cells are unique among somatic cells in that they can activate telomerase after stimulation or during development.^16^, ^17^ T cells stimulated with antigen, mitogen or antibody show increased telomerase activity for around 3 weeks, after which it begins to decline.^16^, ^18^ Experiments with CD8‐positive T cells revealed that these cells can show telomerase activity after two exposures to antigen, and this activity can be extended in the CD4‐positive T cells through seven exposures. Telomerase stimulation in CD4‐ and CD8‐positive T cells requires costimulation of the CD28 surface receptor, which is detected on the majority of CD4‐positive T cells but only a few CD8‐positive T cells.^18^

Under physiological conditions, the proliferation of chondrocytes in cartilage is limited. Cartilage ageing is characterized primarily by a slight reduction in the number of chondrocytes and increased cartilage thinning due to pressure forces and abrasion over time.^19^, ^20^, ^21^ The cartilage of patients with OA has a reduced ability to synthesize structural elements, and the production of tissue‐degrading enzymes is accelerated.^22^ Conversely, in healthy individuals, content of the matrix components remains at a similar level throughout the lifespan.^20^ Chondrocytes of patients with OA are exposed to increased oxidative stress, which causes telomere erosion and instability, leading to premature cartilage ageing.^23^

There is no sex‐associated difference in telomere length (TL) in newborns; however, across the lifespan, telomere shortening is more accelerated in men than in women.^24^, ^25^ Analysis of telomere length in a group of over 100,000 healthy individuals showed that telomeres shorten with age, and this trend is maintained until the age of 75.^26^ The trend reverses in subjects older than 75, and longer telomeres have been found in this age group, suggesting an association between long telomeres and longevity.^26^

The objective of this study was to determine which factors influence leukocyte and cartilage telomere length in OA. We analysed TL in leukocytes and cartilage, since the latter is more severely affected by OA. By comparing relative telomere length in cartilage from severely affected area (anterior surface of the medial femoral condyle) versus a less affected area (lateral femoral condyle) and in leukocytes, we evaluated tissue‐specific impact of OA on telomere length and its correlation with OA severity. Our study offers a new perspective by examining sex differences in telomere attrition of leukocytes and cartilage in OA.

## 2 PATIENTS AND METHODS

### 2.1 OA patients and control group

This study was conducted with the approval of the Institutional Review Board (IRB) at Poznan University of Medical Sciences (PUMS), following the Declaration of Helsinki guidelines. Written informed consent was obtained from all participants. The study included 310 OA patients of European descent aged 49–87 years with primary knee osteoarthritis and 302 control individuals in the same age range.

Each OA patient underwent a detailed clinical and radiological examination before TKA. Joint replacement surgery was performed on all OA patients at the Department of Orthopedics and Traumatology, PUMS. The exclusion criteria were as follows: systemic connective tissue disorders (including rheumatoid arthritis), specific and non‐specific joint inflammation, metabolic and post‐traumatic disorders. Both medical history and radiological data were analysed as integral factors in surgical planning. Radiographs with anterior–posterior and lateral views of both knees were taken in a standing position, and full‐leg radiographs were taken in a vertical position to evaluate limb axis and other joints. The Kellgren‐Lawrence scale was used to grade OA severity based on radiographs.^27^ Pain evaluation in OA patients was performed before TKA using the Knee Society Scores (KSS; 0, 10, 20, 30, 40, 45 and 50) representing severe (0), moderate (10 and 20), mild (30, 40 and 45) pain and none (50).^28^ The extent of articular cartilage damage was also intraoperatively evaluated based on the semi‐quantitative Outerbridge scale.^29^

The control group consisted of individuals selected from the same population of European descent from the same region of Western Poland based on a detailed questionnaire that excluded primary and secondary OA. Weight and height measurements were taken and body mass index (BMI) was calculated in patients and control group. Information about smoking status and a history of depression, reflecting psychological stress, were also collected in both groups.

Peripheral blood was collected from all control individuals and OA patients before TKA. Small cartilage fragments were collected from 57 OA patients during TKA. In each patient, areas with the most advanced OA changes (the anterior surface of the medial femoral condyle) were used for comparison with cartilage samples from the intact area (the anterior surface of the lateral femoral condyle) that served as controls. OA usually progresses from the medial compartment of the knee joint towards the lateral, leaving the latter less affected. We confirmed this trajectory of disease using radiographs in our patients. The macroscopic assessment of cartilage damage was performed during TKA surgery. Cartilage with visible signs of OA such as fibrillation, erosion or thinning was classified as ‘severely affected’. Cartilage appearing visually healthier and less compromised by OA pathology from lateral femoral condyle, was classified as ‘less affected’ and used as ‘control’.

Blood and tissue samples in PBS were transported under refrigeration at 4–8°C to the Department of Medical Genetics, PUMS, for DNA isolation.

### 2.2 Telomere length measurement using qPCR

Genomic DNA was isolated from leukocytes of 612 individuals (310 OA patients and 302 controls) using the salting‐out method. DNA was isolated from 114 cartilage tissue fragments of 57 patients using the DNA Wizard SV Genomic DNA Purification System (Promega). DNA concentration was measured using the Qubit 2.0 Fluorometer (Life Technologies).

Relative telomere length was measured in peripheral blood mononuclear cells (PBMCs) and chondrocytes using a modified quantitative polymerase chain reaction method (qPCR).^30^ In each analysed DNA sample, separate qPCR reactions were performed in triplicate for β‐globin and telomere region. The reaction for telomeric region contained 15 ng of DNA, 1x SYBR Green qPCR Master Mix (Life Technologies), 100 nM Tel‐1b primer (5′‐CGG TTT GTT TGG GTT TGG GTT TGG GTT TGG GTT TGG GTT‐3′) and 900 nM Tel‐2b primer (5′‐GGC TTG CCT TAC CCT TAC CCT TAC CCT TAC CCT TAC CCT‐3′). The reactions were performed in a final volume reaction of 20 μL. The following thermal profile was applied: one cycle of initial denaturation (95°C for 10 min), 30 quantification cycles (95°C for 15 s and 54°C for 1 min) and one melting curve cycle (95°C for 15 s; 60°C for 30 s, and 95°C for 15 s). The β‐globin gene reactions consisted of 15 ng genomic DNA, 1x SYBR Green qPCR Master Mix, 300 nM Hbb3 primer (5′‐TGT GCT GGC CCA TCA CTT TG‐3′) and 700 nM Hbb4 primer (5′‐ACC AGC CAC CAC TTT CTG ATA GG‐3′). The thermal profile included the initial denaturation (95°C for 10 min) followed by 40 quantification cycles (95°C for 15 s, 58°C for 20 s and 72°C for 28 s) and the melting curve cycle (95°C, 15 s; 60°C, 30 s and 95°C, 15 s). The reaction yield in each reaction plate was determined using standard curves (three series of twofold dilutions of reference DNA at concentrations of 60 ng, 30 ng, 15 ng, 7.5 ng and 3.75 ng). Control DNA from CEPH Individual 1347–02 (Life Technologies) was used as a reference for DNA isolated from PBMCs. For chondrocytes, control DNA was obtained from cartilage collected from the knee of a single deceased individual without osteoarthritis. To estimate telomere length, we calculated relative T/S ratio (telomeres/single copy of the β‐globin gene) using the comparative Ct method (2^−ΔΔCt^). Estimated relative telomere length obtained using qPCR measurement is an average TL of all chromosomes; this measurement does not include information about the TL of individual chromosomes.

### 2.3 Data and statistical analysis

Statistical analyses were conducted using relevant functions in the R software package (www.r‐project.org). Factors contributing to the variation of relative telomere length in leukocytes were investigated using a multiple linear regression model, including age, sex (female/male) and disease status (case/control) as the main effect and the interaction between sex and disease status. As age is a potential contributing factor to telomere length, it was included in the regression model as a covariate. Regression analysis was also performed for TL measured in affected and less affected (control) cartilage from the same OA patient, with age and sex being the covariates. The effects of pain in OA patients and of BMI, smoking and depression in OA and control groups were analysed using linear regression models and age/sex as covariates. Differences in TL between tissues (control vs. severly affected cartilage, leukocytes vs. control cartilage and leukocytes vs. severly affected cartilage) were determined using paired *t*‐test. Chi‐squared tests were used to compare Kellgren‐Lawrence scale and Outerbridge scale between male and female patients.

## 3 RESULTS

### 3.1 Collected data: patients and controls

For all parameters in the study, we calculated the mean values for further statistical analysis (Table 1).

**TABLE 1:** Basic characteristics of analysed OA patients and controls (subjects were at least 49 years old).

	OA Patients			Control Group		
	Overall	Females	Males	Overall	Females	Males
Counts	310	233	77	302	129	173
Age	68.4 (8.0)	69.0 (7.7)	66.7 (8.7)	65.3 (7.6)	66.8 (8.3)	64.2 (6.8)
BMI	31.9 (4.8)	32.3 (4.8)	31.0 (4.9)	29.4 (5.2)	29.5 (5.9)	29.3 (4.6)
Relative TL in leukocytes	1.87 (0.59)	1.81 (0.56)	2.09 (0.63)	2.21 (0.60)	2.17 (0.56)	2.24 (0.62)
Smoking status	286	216	70	270	112	158
Smoker	26 (9.1%)	12 (5.6%)	14 (20%)	87 (32.2%)	34 (30.4%)	53 (33.5%)
Non‐Smoker	260 (90.9%)	204 (94.4%)	56 (80%)	183 (67.8%)	78 (69.6%)	105 (66.5%)
History of depression	288	218	70	270	112	158
Yes	32 (11.1%)	30 (13.8%)	2 (2.9%)	34 (12.6%)	13 (11.6%)	21 (13.3%)
No	256 (88.9%)	188 (86.2%)	68 (97.1%)	236 (87.4%)	99 (88.4%)	137 (86.7%)
KSS Pain Scale^b^	310	233	77			
None 50	0 (0%)	0 (0%)	0 (0%)			
Mild or occasional 45	1 (0.3%)	1 (0.4)	0 (0%)			
Mild (stairs only) 40	25 (8.1%)	17 (7.3%)	8 (10.4%)			
Mild (walking & stairs) 30	95 (30.6%)	75 (32.2%)	20 (26%)			
Moderate ‐ Occasional 20	119 (38.4)	84 (36.1%)	35 (45.4%)			
Moderate ‐ Continual 10	52 (16.8%)	41 (17.6%)	11 (14.3%)			
Severe 0	18 (5.8%)	15 (6.4%)	3 (3.9%)			
Relative TL in less affected cartilage^a^	1.11 (0.84)	1.01 (0.75)	1.44 (1.10)			
Relative TL in severly affected OA cartilage^a^	0.76 (0.66)	0.69 (0.54)	1.02 (0.96)			
Outerbridge Scale^b^	1: 2 (0.6%)	1: 1 (0.4%)	1: 1 (1.3%)			
	2: 20 (6.5%)	2: 14 (6.0%)	2: 6 (7.8%)			
	3: 92 (29.7%)	3: 67 (28.8%)	3: 25 (32.5%)			
	4: 196 (63.2%)	4: 151 (64.8%)	4: 45 (58.4%)			
KL Scale^b^	2: 52 (16.8%)	2: 35 (15.0%)	2: 17 (22.1%)			
	3: 156 (50.3%)	3: 117 (50.2%)	3: 39 (50.6%)			
	4: 102 (32.9%)	4: 81 (34.8%)	4: 21 (27.3%)			

*Note*: Values shown (except for ‘Counts’, ‘Smoking Status’, ‘History of depression’, ‘KSS Pain’ ‘Outebridge Scale’ and ‘KL Scale’) are means with the corresponding standard deviations in parentheses.

^a^ Relative telomere lengths are calculated based on 57 patients (45 females and 12 males).

^b^ Within each cell, the numerical values indicate the scale value, the count of a specific scale value and the corresponding percentage within the corresponding group.

### 3.2 Relative TL in leukocytes of OA patients and control group

Analysis of relative TL in leukocytes of healthy individuals and patients with osteoarthritis showed that age, sex and disease status are all significant contributors to the variation of relative TL with the following fitted model:

Relative TL in leukocytes = 2.332–0.00763*Age + 0.269*Sex ‐ 0.347*Status +0.217*Sex*Status, with the following coding schemes: for Sex, female and male were coded as 0 and 1, respectively; and for Status, control and case were coded as 0 and 1, respectively. The fitted regression model has an *R* ^2^ value of 0.104, meaning it can explain about 10.4% variation of the relative TL in leukocytes. When other factors are adjusted, increasing age results in shorter telomere lengths. We found that a one‐year increase in age corresponds to a 0.00763 relative TL unit reduction in telomere length.

**FIGURE 1:**
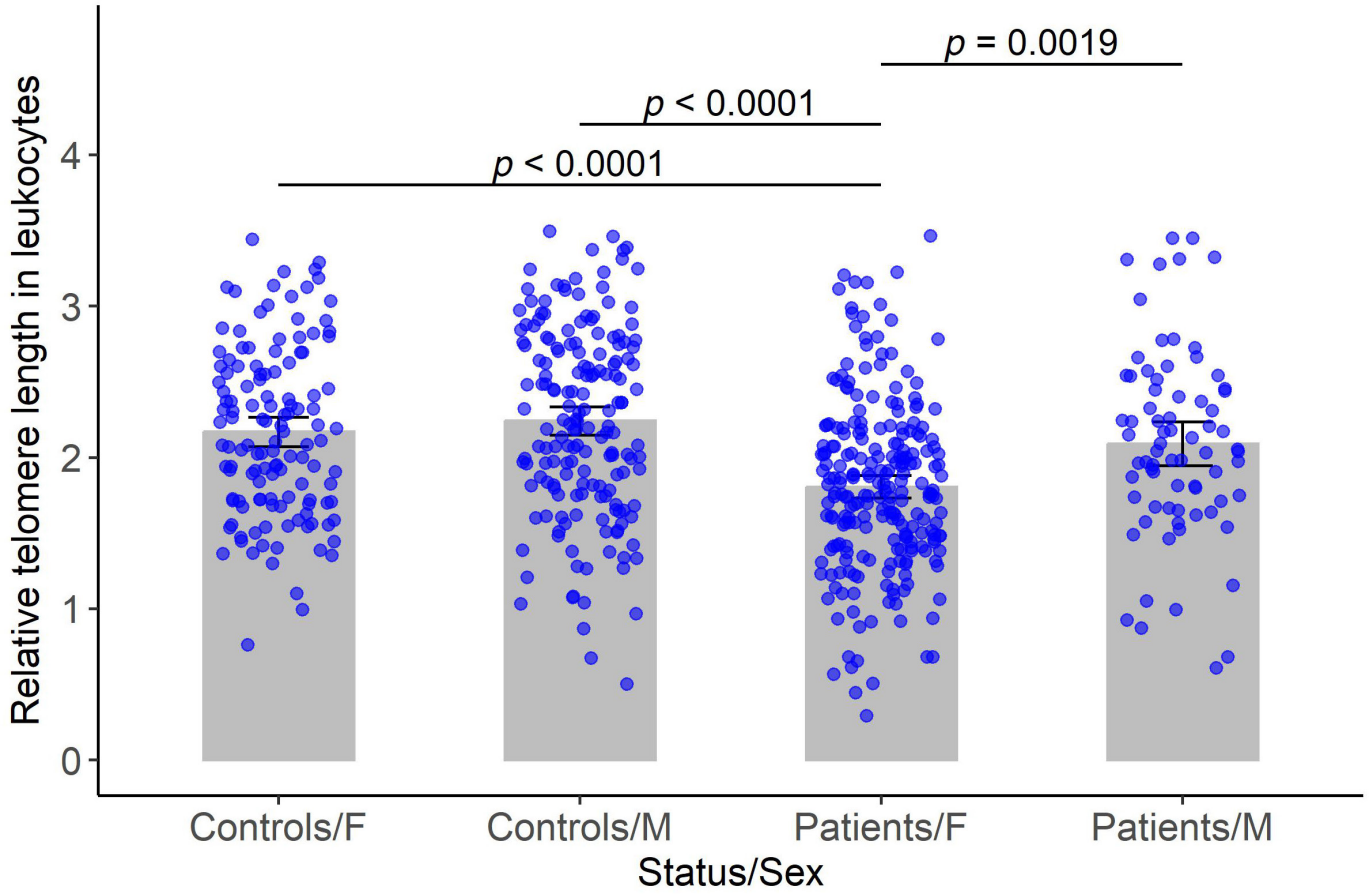
Relative TL in leukocytes in different status/sex groups. The top of each bar represents the mean of the relative telomere length for the corresponding status and sex category, and the vertical lines represent the 95% confidence interval of the mean. Note that comparisons were conducted for all possible pairs, with Bonferroni adjusted *p*‐values less than 0.05 indicated in the plot.

When controlling for age, relative TL of male controls averaged 0.269 (95% confidence interval [CI] [0.115, 0.423], *p*‐value = 0.000654) longer than that of female controls (Figure 1), while female patients with osteoarthritis on average had 0.347 (95% CI [0.219, 0.474], *p*‐value = 1.24*10^−7^) shorter relative telomere length than female controls, and male patients on average had 0.486 (95% CI [0.352, 0.621], *p*‐value = 0.00232) longer TL than female patients.

A significant sex‐by‐disease‐status interaction was found in regard to relative telomere length: When controlling for age, the average reduction in TL between female patients and controls was 0.217 (95% CI [0.014, 0.421], *p*‐value = 0.0367) greater than the reduction in TL between male patients and controls (Figure 1).

### 3.3 Comparison of relative TL in cartilage and leukocytes

**FIGURE 2:**
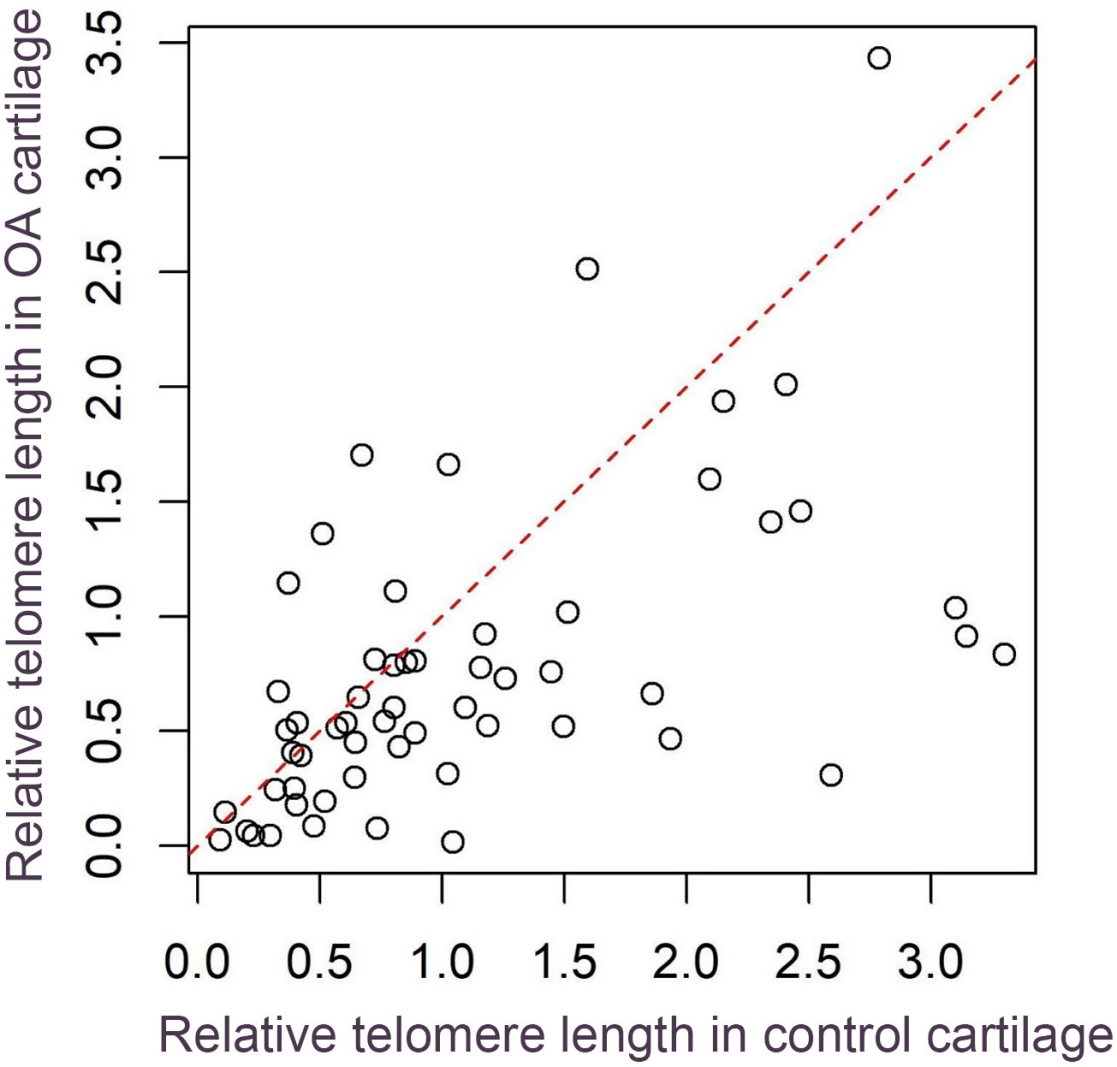
Relative telomere length in OA and control cartilage samples collected from the same knee in each of 57 patients. Points below the dashed line (y = x) represent individuals with longer relative TL in control cartilage than in OA cartilage. Note that 'OA cartilage' refers to severly affected cartilage collected from the medial femoral condyle, while 'control cartilage' refers to less affected cartilage from the lateral femoral condyle.

**FIGURE 3:**
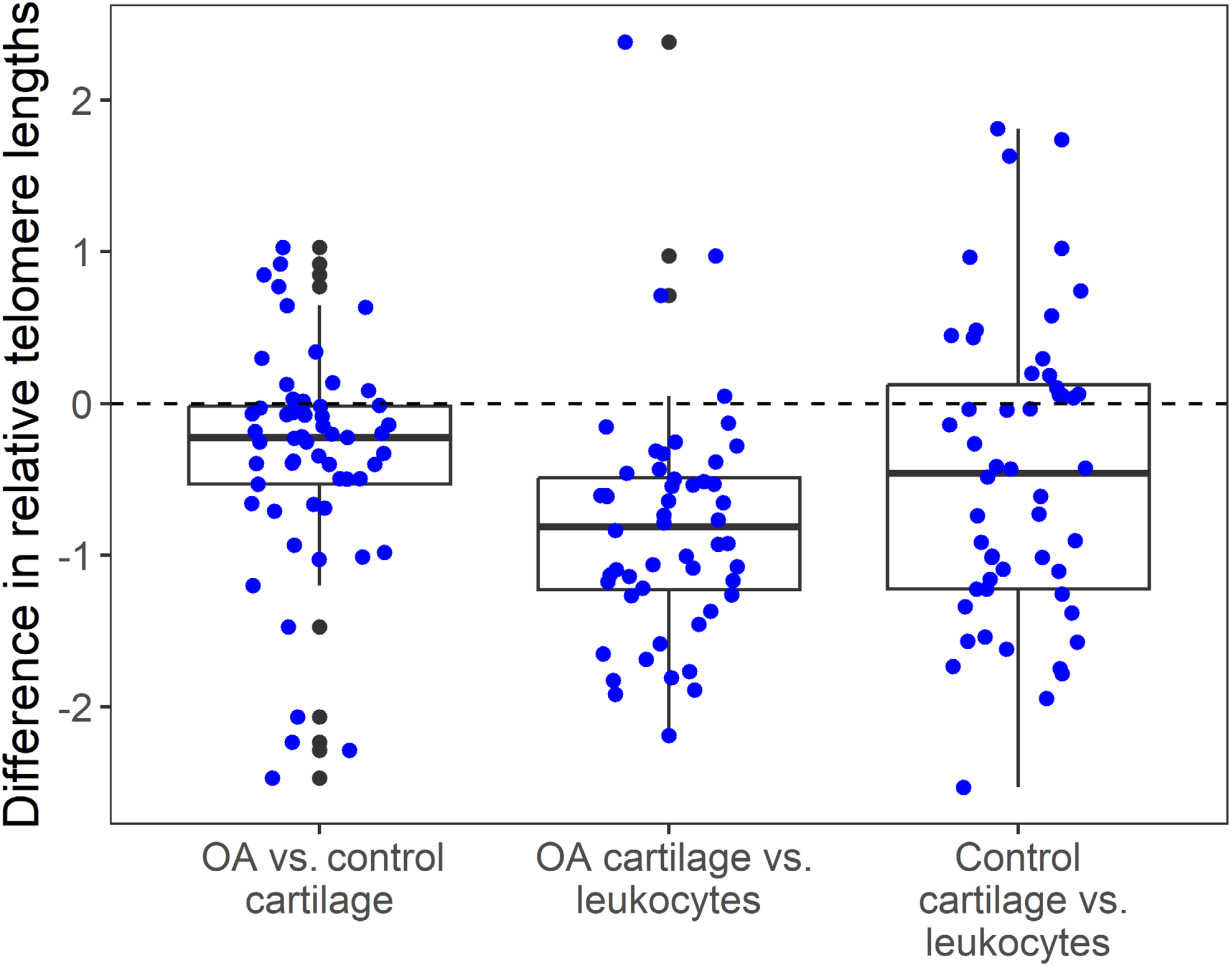
Pairwise comparisons of relative TL differences in cartilage and leukocytes of 57 patients with OA. Comparisons were performed using paired *t*‐tests, with Bonferroni adjusted *p*‐values. Y axis is the difference in relative TL and the comparisons shown on the X axis are as follows: (1) OA versus control cartilage (*p*‐value = 7.45*10^−4^); (2) OA cartilage versus leukocytes (*p*‐value = 8.50*10^−10^; (3) control cartilage versus leukocytes (*p*‐value = 0.0014). The horizontal dashed line represents y = 0. Each boxplot represents the difference in relative TL for a selected comparison; the bold line in the middle of the box is the median of the distribution.

Relative telomere length measurements in affected and less affected (control) cartilage samples obtained from the same joint of each OA patient (*n* = 57; 45 women and 12 men) during TKA (Figure 2) were compared using paired *t*‐tests, with Bonferroni adjusted *p*‐values (Figure 3). Relative TL in affected cartilage was shorter than control cartilage (*p* = 7.45*10^−4^), 0.345 mean difference with 95% CI [0.151, 0.539]), (Figure 3). We also compared relative TL in cartilage collected from severely affected and less affected parts of each joint with TL in leukocytes of these patients. Relative TL in severly and less affected cartilage samples was shorter than relative TL in leukocytes (*p* = 8.5*10^−10^ and 1.4*10^−3^ respectively, mean differences with 95% CI of 0.810 [0.594, 1.027] and 0.464 [0.189, 0.739], respectively), (Figure 3).

Regression analysis of relative TL in cartilage samples of patients with osteoarthritis showed that sex is a significant contributor to the variation in relative TL when age is controlled, with the following fitted model:

$$\text{Relative}\;\mathrm{TL}\;\text{in}\;\mathrm{OA}\;\text{cartilage}=-0.5887+0.4370^{*}\mathrm{Sex}+0.0180^{*}\mathrm{Age}$$

The coding scheme for sex was the same as previous analyses: female and male were coded as 0 and 1, respectively. The fitted regression model has an *R* ^2^ value of 0.102, meaning it can explain about 10.2% of the variation in relative TL in severely affected cartilage by OA. When controlling for age, relative TL in this tissue from male patients was on average 0.4370 (95% CI [0.084, 0.865]; *p* = 0.0458) longer than that of female patients. A similar analysis of less affected (control) cartilage from OA patients revealed no evidence of a sex difference (average 0.5280 with 95% CI [−0.029, 1.085]; *p* = 0.0627).

### 3.4 Pain and telomere length in leukocytes and cartilage of patients with OA

**FIGURE 4:**
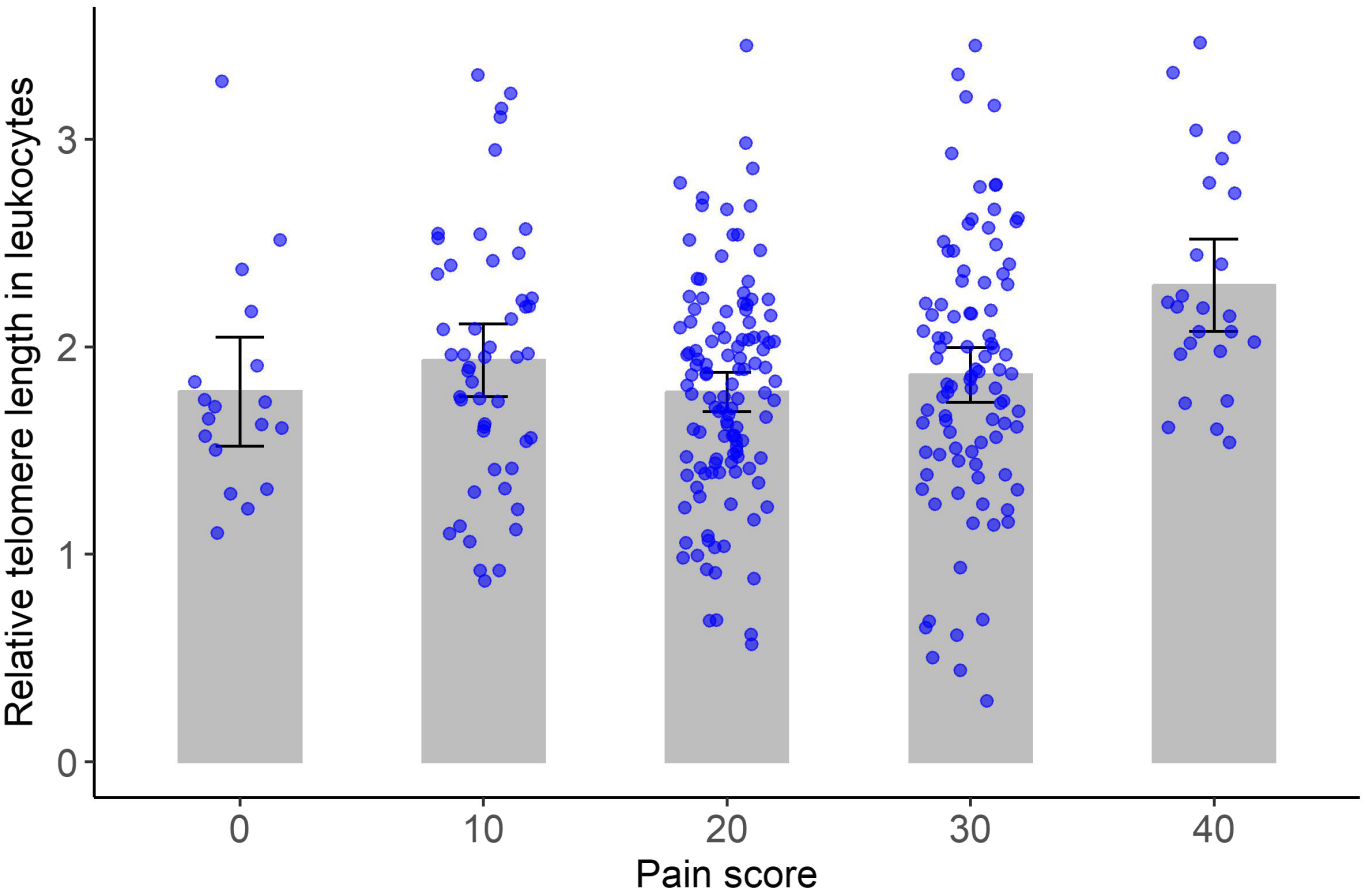
Relative telomere length in OA patients grouped according to their pain level. Pain was estimated with KSS scores, representing severe‐to‐mild pain (0, 10, 20, 30 and 40). The top of each bar represents the mean of the relative telomere length for the corresponding pain score, and the vertical lines represent the 95% confidence interval of the mean.

A significant relationship was detected between pain and relative leukocyte telomere length in OA patients when adjusted for age and sex (*F*‐statistic = 4.111, degrees of freedom of 4 and 300, *p* = 0.002944). In general, OA patients with mild pain (KSS 40) had longer leukocyte telomeres than patients with higher pain scores (KSS 0, 20 or 30; Figure 4). TL for patients with a KSS score of 30 was an average of 0.3970 TL units shorter than TL for those with a score of 40, when adjusted for age and sex (Figure 4).

A similar analysis was performed in affected and control cartilage of OA patients; however, no association between pain and telomere length was detected.

## 4 DISCUSSION

Despite its high prevalence, the aetiology of primary osteoarthritis remains poorly understood. The aim of our study was to determine whether telomere length of leukocytes and chondrocytes of patients with primary knee OA is significantly affected by the disease and factors like pain, depression, smoking and male or female sex.

Our results show that age, sex and disease status are all important factors contributing to leukocyte TL. Relative TL in leukocytes reduces by 0.00763 units for each year of age. Female patients with OA show a greater relative TL reduction versus controls in leukocytes compared to male patients versus controls (Figure 1). Telomeres in the severely affected cartilage of female OA patients are shorter than those in male patients, which suggests that sex‐associated TL differences in OA are not confined to leukocytes alone. These results may indicate that factors contributing to telomere attrition are intensified or more complex in female patients compared with male patients (Figure 1). OA is more prevalent in women than in men; however, sex‐associated differences in relative TL of leukocytes and cartilage have not yet been reported in this disease.^31^ Oestrogen has well‐documented anti‐inflammatory properties at the systemic and tissue levels. It protects cartilage from reactive oxygen species (ROS) via inhibition of cyclooxygenase‐2 (COX‐2) expression.^32^, ^33^ A drop in oestrogen level is accompanied by high ROS production, activating expression of nuclear factor ĸB (NF‐ĸB) and downstream pro‐inflammatory cytokines.^34^

The female OA patients in our study were in menopausal and postmenopausal age ranges, meaning that they were exposed to increased ROS level due to both oestrogen depletion and low‐level inflammation caused by OA. Both factors may contribute to the more severe TL loss we observed compared to men with primary OA.

In a cohort of 80 patients with knee OA, Poonpet et al. reported increased telomere shortening in leukocytes compared to 60 healthy controls.^35^ In 2019, Mosquera et al. reported that TL in leukocytes is a risk factor for concurrent knee OA and has a negative correlation with age, BMI and waist circumference.^36^

Sibille et al. found that patients affected by chronic pain and stress associated with knee OA have significantly shorter telomeres than patients without pain.^37^, ^38^ Our results indicate that patients experiencing mild pain while using stairs (score 40) exhibited longer leukocyte TL compared to patients with severe (score 0), moderate (score 20) and mild pain during both walking and using stairs (score 30), (Figure 4). A recent study by Guillen et al. indicates an association between telomere loss in leukocytes and development of incident radiographic knee OA.^39^

In our study, we compared the relative telomere length of chondrocytes collected from severely affected areas and less affected areas of OA joints from the same knee in 57 patients (Figures 2 and 3). We observed significant telomere shortening in cartilage collected from the area of the joint with highest damage compared to distal, less affected parts of the knee joint (*p* = 7.45*10^−4^), (Figure 3). In a previous study of three patients with knee OA, researchers utilized Universal STELA and Q‐FISH methods to evaluate differences in chondrocyte TL from sites of varying distance from the center of OA changes within the same knee joint.^40^ Results showed a decrease in TL in areas of the joint with the most severe damage.^40^

Our results show that both more affected and less affected (control) chondrocytes collected from the knees of OA patients have shorter telomeres compared to leukocytes of the same patients (*p* = 8.5*10^−10^ and 1.4*10^−3^) (Figure 3). This result contradicts prior observations of Tamayo et al., who used qPCR to analyse TL in 24 patients with knee OA and reported that OA telomeres in chondrocytes were longer than those in leukocytes.^41^ Tamayo et al. also reported increased genomic instability in OA patients, manifested by numerical chromosome aberrations in chondrocytes and peripheral blood leukocytes. This analysis was performed using fluorescence in situ hybridization (FISH) with probes specific to autosomes 7, 8, 18 and both sex chromosomes. Chondrocytes from OA patients showed more aberrations than chondrocytes from healthy controls.^41^ The largest group of identified aberrations were aneuploidies, mainly monosomies and trisomies. Chondrocytes of OA patients also revealed a higher percentage of numerical aberrations than peripheral blood leukocytes from the same patient; for example, the incidence of trisomies was established as 3.9:1.^41^

Our results support prior studies suggesting that mechanical stress and ageing of joints lead to substantial release of reactive oxygen species (ROS), which damages cartilage telomeres and increases the number of ultrashort telomeres.^40^ Cartilage injury enhances proliferation of chondrocytes in joints by severalfold, contributing to gradual telomere shortening with fewer ultrashort telomeres.^40^, ^42^ Both gradual and stochastic models seem to overlap in the aetiology of OA^40^ and result in severe telomere shortening in cartilage despite a naturally low proliferation rate of chondrocytes. These two overlapping processes in knee cartilage may lead to accelerated telomere shortening in chondrocytes to a degree that is more severe than in leukocytes. Consequently, telomere length measurements in leukocytes of OA patients should not be used to infer telomere length in cartilage. We found that women with OA exhibited shorter telomeres in both leukocytes and severely affected cartilage compared to men, suggesting that there may be intensified factors contributing to TL reduction in female patients. Sex‐associated differences were not detected in Outerbridge and Kellgren‐Lawrence scales in OA patients (Table 1).

We also observed that increased pain is associated with shorter TL in leukocytes, but not in cartilage. This highlights tissue‐specific differences in TL linked to OA. Notably, this association was not linear: In our analysis, only OA patients with mild pain, as indicated by a KSS score of 40, showed significantly longer leukocyte TL compared to those with higher pain (KSS 30, 20 or 0, (Figure 4). This result suggests that a broad spectrum of pain affects telomere length in leukocytes of OA patients.

Depression (as an indicator of psychological stress) and smoking had no effect on TL of leukocytes and cartilage.

## AUTHOR CONTRIBUTIONS

**Malwina Czarny‐Ratajczak:** Conceptualization (lead); formal analysis (equal); funding acquisition (lead); investigation (equal); methodology (lead); project administration (lead); resources (lead); supervision (lead); writing – original draft (lead); writing – review and editing (lead). **Lukasz Kuszel:** Formal analysis (supporting); funding acquisition (supporting); investigation (lead); project administration (equal); writing – original draft (equal). **Tomasz Trzeciak:** Conceptualization (supporting); data curation (equal); funding acquisition (supporting); investigation (equal); resources (equal); writing – original draft (equal). **Beata Begier‐Krasinska:** Data curation (equal); investigation (equal). **Magdalena Richter:** Data curation (supporting); investigation (equal); writing – original draft (equal). **Jian Li:** Conceptualization (supporting); data curation (lead); formal analysis (lead); software (lead); validation (lead); visualization (lead); writing – original draft (equal).

## FUNDING INFORMATION

Funding sources played no role in the study design, data collection, analysis, manuscript preparation or decision to publish.

## CONFLICT OF INTEREST STATEMENT

The authors declare no conflict of interest.

## INSTITUTIONAL REVIEW BOARD STATEMENT

The study was conducted in accordance with the guidelines of the Declaration of Helsinki and approved by the Institutional Review Board at Poznan University of Medical Sciences (IRB approvals: 370/11 and 1158/12) and Tulane University (IRB approval #140391).

## INFORMED CONSENT STATEMENT

Written informed consent was obtained from all subjects involved in the study.

## Data Availability

The data that support the findings of this study are available on request from the corresponding author. The data are not publicly available due to privacy or ethical restrictions.
